# The effect of a specific vitamin supplement containing L-methylfolate (Ocufolin forte) in patients with neovascular age-related macular degeneration

**DOI:** 10.1016/j.aopr.2025.03.001

**Published:** 2025-03-04

**Authors:** Tatjana Josifova, Katarzyna Konieczka, Andreas Schötzau, Josef Flammer

**Affiliations:** a Eye Clinic Orasis, Department for Medical and Surgical Retina, Reinach, Switzerland; b Department of Ophthalmology, University of Basel, Switzerland; c Glaucoma Eye Practice Dr. K. Konieczka, Basel, Switzerland; d Statistical Consulting, University of Basel, Switzerland

**Keywords:** Neovascular age-related macular degeneration, Retinal venous pressure, Homocysteine, Ocufolin forte, Anti-VEGF therapy, OCT, OCTA

## Abstract

**Background:**

Patients with nAMD often have pathologically elevated homocysteine (Hcy) and increased retinal venous pressure (RVP). We tested whether the administration of a specific vitamin preparation containing L-methylfolate (Ocufolin forte) as an addition to anti-VEGF therapy reduces these two risk factors and favorably influences the disease course.

**Methods:**

A total of 27 eyes/27 patients with intra- and subretinal fluid, Hcy above 12 ​μmol/L, RVP of at least 8 ​mm above the IOP, and an IOP between 10 and 20 ​mmHg were included in this study. All eyes received three injections of 0.05 ​ml aflibercept at one-month intervals as clinically indicated. Fifteen patients additionally received one capsule of Ocufolin forte per day (Ocufolin group, OG), and the other twelve patients served as a control group (control group, CG). The following factors were measured before therapy and four months later: blood Hcy, best-corrected visual acuity (BCVA), intra-ocular pressure (IOP), RVP, optical coherence tomography (OCT), and optical coherence tomography - angiography (OCTA).

**Results:**

Hcy decreased on average by 5.58 ​μmol/L in the OG and by 0.57 ​μmol/L in the CG. The RVP decreased on average by 4.60 ​mmHg in the OG and by 0.75 ​mmHg in the CG. The difference between the two groups was significant for both parameters (*P* ​<0.001); 66% of the OG and 41% of the CG had no retinal fluid at the end of the study. After the completion of the study, the injection intervals could be extended more often in the OG patients than in the CG patients.

**Conclusions:**

When Ocufolin forte was added to the standard therapy, RVP and Hcy were reduced to a significantly greater extent than without Ocufolin forte. In addition, Ocufolin had a positive influence on morphology and future treatment intervals with anti-VEGF therapy.

## 1 Introduction

*Age-related macular degeneration* (AMD) significantly reduces the quality of life of affected patients and is a major socio-economic burden on society due to its high prevalence.^1^ The disease leads to a decrease in central vision due to extracellular deposits, pigment epithelium changes, and degeneration of the photoreceptors.^2^ At a later stage, this condition, known as dry AMD (dAMD) can lead to outer retina atrophy (geographic atrophy, GA) or to neovascularization (nAMD). The pathophysiology is complex and only partially known; however, hypoxia and oxidative stress and inflammation certainly play a role.^3^ The therapeutic options for this condition are still limited. While the focus of research in nAMD is on anti-VEGF therapy,^4^ antioxidants and complement inhibitors are used to treat dAMD.^1^ The risk factors (RFs) for these conditions are fairly well known. Interestingly, the RFs for cardiovascular diseases such as hypercholesterolemia, arterial hypertension, or smoking are also RFs for AMD. One of these RFs is elevated Hcy.^5^^,^^6^ Another RF that is still poorly known and little researched is increased retinal venous pressure (RVP). The hypoxic macula and its surrounding tissue produce increased levels of hypoxia-inducible factor HIF-1α, a gene expression factor of VEGF and endothelin (co-expression).^7^ While the increase in VEGF leads to neovascularization, endothelin increases the RVP, among other things.^8^ In the present study, we tested whether and how well we can reduce elevated Hcy and RVP (measured with the ophthalmodynamometer according to Löw) in patients with nAMD.

*What is the retinal venous pressure (RVP) and why is it relevant?* The blood flow in the retina is determined by the perfusion pressure (PP) and the vascular resistance.^9^ The PP is defined as the difference between the arterial pressure at the entrance to the eye and the RVP at the exit of the eye. If the arterial blood pressure drops or the RVP rises, the PP falls. If this can no longer be compensated by autoregulation, the blood flow decreases.^10^ In the past, it was assumed that the RVP was the same as the IOP, except in the case of high intracranial pressure (ICP).^11^ Today, we know that, although the RVP in healthy people is usually equal to the IOP, the RVP often significantly exceeds the IOP in certain eye diseases such as diabetic retinopathy^12^ or glaucoma^8^ and in both eyes of patients with unilateral retinal vein occlusion^13^ as well as in some systemic conditions such as Flammer syndrome.^14^ The RVP is generally elevated in patients with elevated endothelin-1.^8^ A high RVP not only lowers the PP but also increases the transmural pressure in the capillaries. Together with the simultaneous weakening of the blood–retina barrier by endothelin, this leads to increased fluid extravasation.^15^^,^^16^ As the retina has no lymphatic vessels, fluid must be reabsorbed from the extravascular space back into the blood vessels. In other words, if the transmural pressure rises or the oncotic pressure gradient falls, e.g., due to a weak or defective blood–retinal barrier, edema will result in the retina. The measurement of the RVP has evolved over the last few decades.^10^ However, all methods are based on the observation of retinal venous pulsation. As the IOP is normally higher than the ICP, the venous blood leaves the eye along a more or less steep pressure gradient. The size of this gradient fluctuates synchronously with the heart rhythm because the pulsation of the IOP is slightly out of phase with the pulsation of the ICP. This leads to a rhythmic change in the local flow velocity and, thus, also in the volume of the blood column in the veins in or near the optic disc.^17^ Clinicians can observe this as retinal venous pulsation. If the vein pulsates spontaneously, then the local RVP corresponds to the IOP. If no pulsation is visible, the IOP is artificially increased until the vein begins to pulsate. This additional increase in pressure is called venous incremental pressure (VIP).^18^ The RVP corresponds to the sum of the IOP and VIP.

*What is homocysteine (Hcy) and why is it important?* Hcy is an amino acid that is not incorporated into proteins, but is essential in C1 metabolism. In C1 metabolism, methyl groups are transferred to various molecules and, thus, form the basis of vital substances produced by the body such as hormones, neurotransmitters, and DNA. It is also involved in DNA methylation and thus plays a role in epigenetics, among other things. Methionine, which is obtained via the diet, can release a methyl group, thus becoming Hcy. Hcy in turn can take up a methyl group from L-5-methyltetrahydrofolate (L-methylfolate, 5-MTHF), also called vitamin B9. This reaction requires vitamin B12 and zinc as cofactors. Hcy can also be converted into the homologous cysteine in the presence of vitamin B6 (cysteine is a precursor of the antioxidant glutathione). An increase in Hcy is therefore an indirect sign of L-methylfolate and/or vitamin B12 deficiency. A high Hcy level in the blood is associated with various diseases, especially cardiovascular diseases, but also eye diseases^19^ such as glaucoma^20^ or AMD.^19^^,^^21^^,^^22^ Besides higher Hcy, a reduced vitamin B12 plasma level has also been reported in AMD patients.^22^ The Aliénor study, a cohort study of older adults, showed that normal serum folate levels and high dietary B vitamin intakes are associated with a reduced risk of developing advanced AMD.^23^ A meta-analysis of 11 studies showed that plasma Hcy levels were elevated by 2.67 ​μmol/L on average in AMD patients.^22^ While the statistical context is well documented, there has long been doubt as to whether Hcy is also causally involved in disease development. However, there is much evidence that Hcy may also be directly involved in the pathogenesis of diseases such as AMD.^24^ One hypothesis assumes that Hcy contributes to AMD by triggering metabolism in the mitochondria, in which cells predominantly produce energy through a high rate of glycolysis.^6^ This increased glycolysis leads to elevated lactate production and cellular acidosis, which, in turn, contributes to the neovascularization and dysfunction of the outer blood–retinal barrier.

*What is Ocufolin*? We now know that patients with degenerative eye diseases have a greater need for certain micronutrients.^25^^,^^26^ Ocufolin contains these very components. The main ingredient is the circulating form of the bioactive folate: L-methylfolate. The formulation also contains other B vitamins that help to reduce elevated Hcy levels. In addition, its lipophilic matrix contains minerals and antioxidants that were proven useful in the ARED studies.^27^ The recommended dose for patients with AMD is one capsule of Ocufolin forte a day. Ocufolin has already been shown to lower Hcy in diabetic patients.^28^ Devogelaere et al. have shown that Ocufolin forte is very effective in reducing both Hcy and the RVP in glaucoma patients.^29^

In this study, we investigated whether the addition of Ocufolin forte to the standard therapy of patients with neovascular AMD additionally reduces RVP and Hcy and possibly has an additional effect on morphology and/or VA.

## 2 Patients and methods

Patients who were scheduled to receive intravitreal injections (IVIs) based on clinical criteria and who met the following criteria: nAMD with CNV type 1, 2 or 3, subretinal or intraretinal fluid and a foveal thickness of 300 ​μm or more, an RVP of at least 8 ​mmHg above IOP and an Hcy level of 12 ​μmol/L or more were eligible for the study. These patients were informed that they could voluntarily participate in a study in which about half of the patients would receive additionally one capsule per day of a commercially available vitamin preparation called Ocufolin forte for four months.^30^ They were informed that Ocufolin has been registered in Switzerland (and some other countries) for several years as a food for special medical purposes (FSMP). Patients who decided to participate in the study gave their written consent.

Of the 29 patients who decided to participate, 15 were randomly allocated to the Ocufolin group (OG) and 14 to the control group (CG).

Two patients in the CG had to stop receiving the anti-VEGF injections for general medical reasons and therefore dropped out. Fifteen patients in the OG and twelve in the CG completed the study. All patients had previously received multiple intravitreal anti-VEGF injections (IVIs); see Table 1. During the study, both groups received three standard injections of 2 mg/0.05 ​ml aflibercept (Eylea®) at one-month intervals. Patients in the OG additionally received one capsule of Ocufolin forte per day.

**Table 1:** Demographics of the study population.

	N of eyes	N of patients	Gender m/f	Age median	N of previous IVIs	nAMD type 1	nAMD type 2	nAMD type 3	Subretinal fluid	Intraretinal fluid	Sub- and Int- fluid
OG	15	15	7/8	77.2	9–23	5	9	1	5	9	1
CG	12	12	9/3	75.8	6–18	3	8	1	7	5	0

Patients in the study did not undergo any additional examinations along with our standard examinations for patients with therapy-refractory nAMD, which included VA, IOP, biomicroscopy of the fundus, OCT, OCTA, RVP (measured with the ophthalmodynamometer according to Löw), and plasma Hcy. For study purposes, the results of these examinations were anonymized and statistically analyzed at the beginning and after 4 months.

In patients with persistent intraretinal or subretinal fluid of ≥80 ​μm on OCT or a loss of ≥5 letters on the Snellen chart at the end of the 4-month study, treatment with IVIs was continued.

Descriptive statistics are presented as mean (SD). OG and CG were statistically compared with the Welch *t*-test using baseline subtracted post-values. Welch *t*-test allows for different variances in the study groups. The results are reported as the mean differences between study groups with corresponding p-values. A *P*-value <0.05 is considered significant. All the evaluations were performed using R version 4.4.0.^31^ Because the sample size was relatively small, we also compared the groups using a non-parametric test (Kruskal-Wallis test).

## 3 Results

The demographic data of the patients included in the study are listed in Table 1. At the beginning of the study, the Hcy, RVP, VA, and IOP were not significantly different between the two groups.

**Fig. 1:**
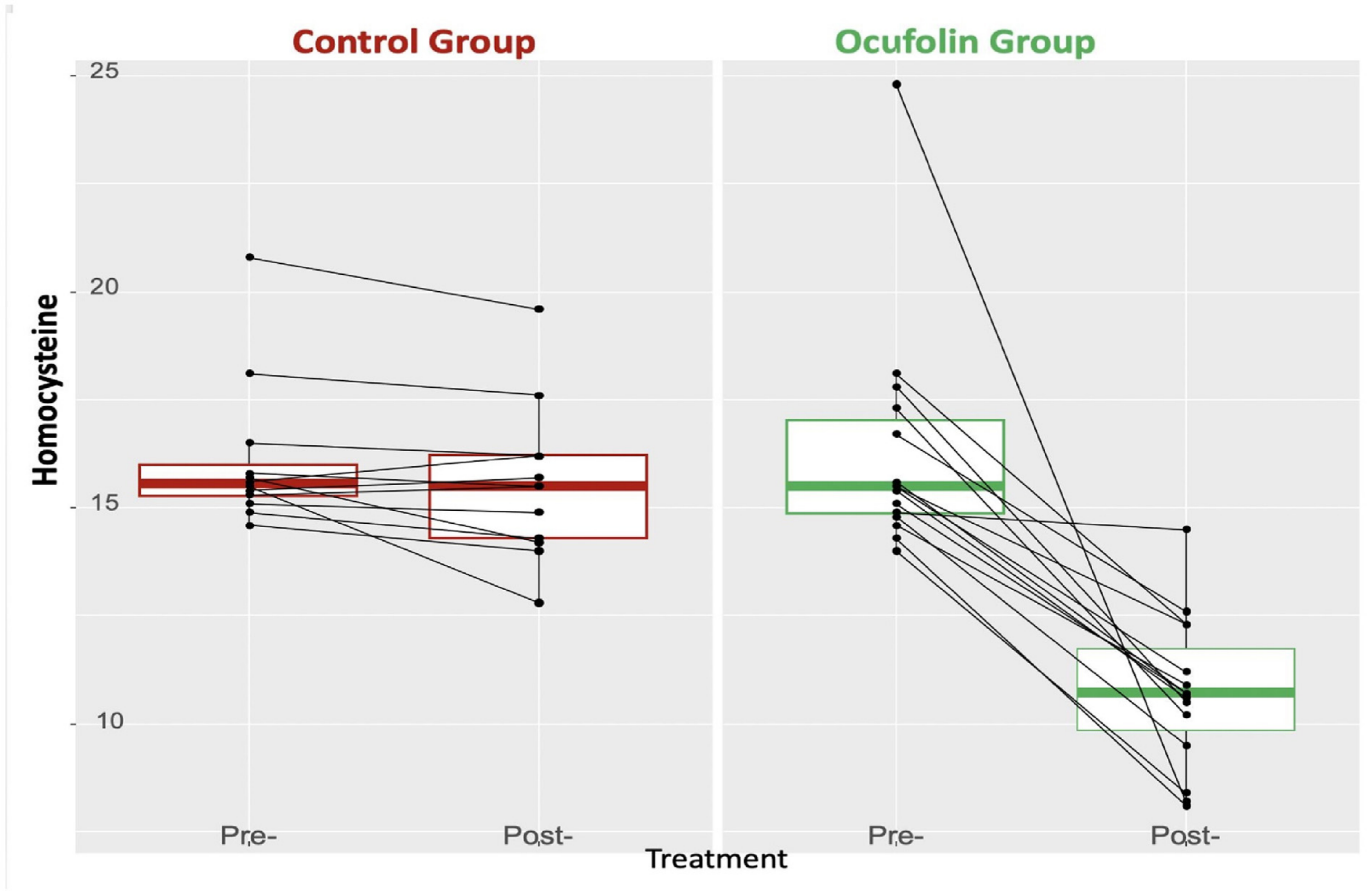
Homocysteine values expressed in μmol/L in the two groups at the beginning and at the end of the study.

The Hcy values before the study and after four months are shown in Fig. 1. Hcy decreased substantially in the OG while it remained virtually unchanged in the CG. The difference between the two groups was significant (*P* ​<0.001); see Table 2.

**Table 2:** Comparisons of differences between the groups, expressed as mean (SD). The groups were compared using both the Welch *t*-test and the Kruskal-Wallis test. P-values of the Kruskal-Wallis test are indicated in brackets.

	ALL	Control Group	Ocufolin Group	*P*-values overall	N
HCY diff	−3.35 (3.65)	−0.57 (0.89)	−5.58 (3.48)	<0.001 (<0.001)	27
RVP diff	−2.89 (2.39)	−0.75 (1.71)	−4.60 (1.12)	<0.001 (<0.001)	27
Visus diff	0.12 (0.11)	0.09 (0.09)	0.14 (0.12)	0.287 (0.397)	27

**Fig. 2:**
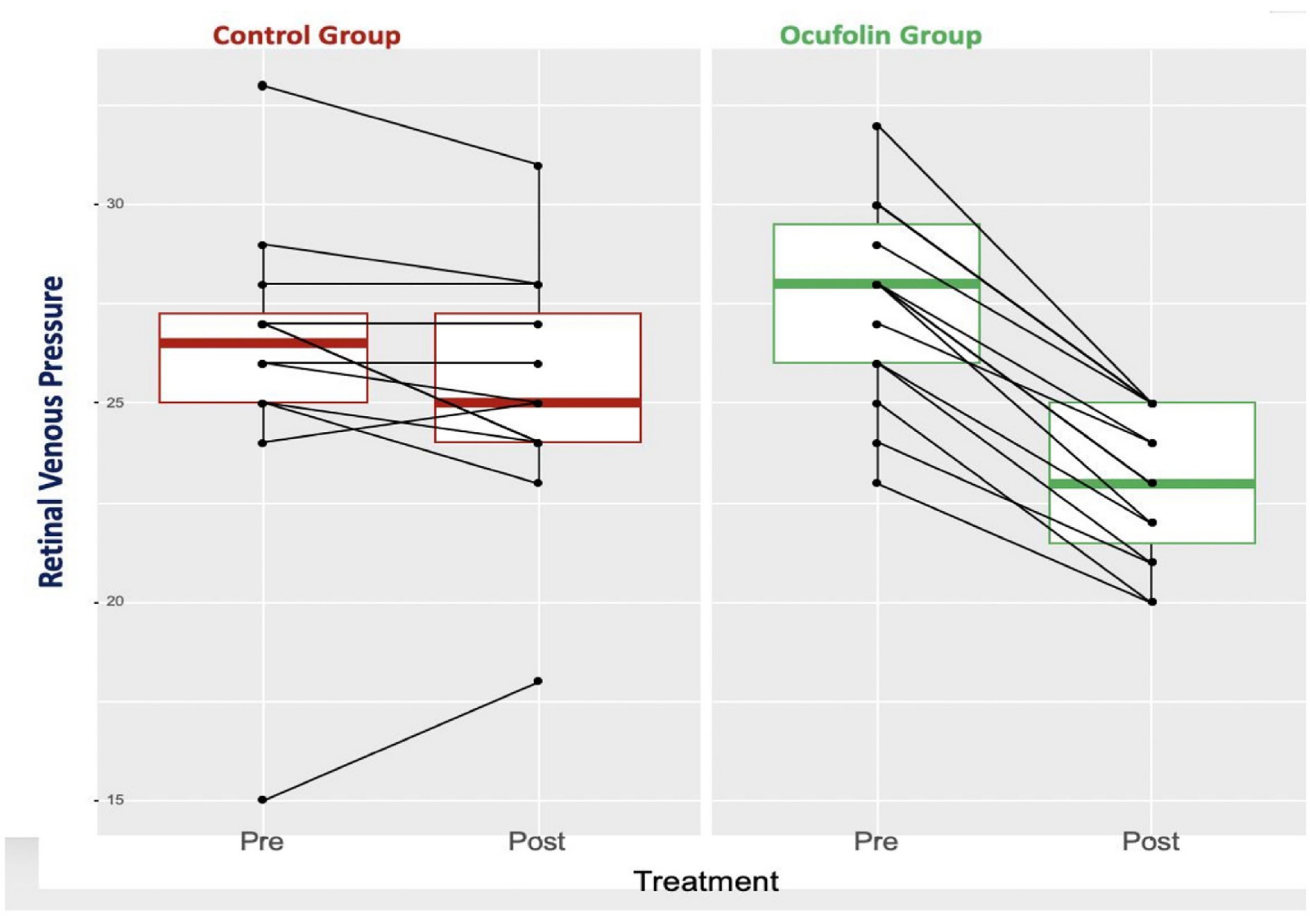
Retinal venous pressure expressed in mmHg in the two groups at the beginning and at the end of the study.

RVP decreased slightly in the CG and substantially in the OG (Fig. 2). The difference between the two groups was significant (*P* ​<0.001); see Table 2. This means that Ocufolin considerably lowered the pathologically elevated RVP, whereas the injection of anti-VEGF alone had only a minor effect on RVP.

In all the patients, the IOP at the beginning of the study and during the study was in the normal range (10–20 ​mmHg), and the mean IOP remained at 15 ​mmHg in both groups. On average, VA improved slightly over the course of the study, and a little more in the OG than in the CG. However, the difference between the two groups was not statistically significant (Table 2). At the end of the study, patients in the OG had less retinal fluid than patients in the CG (Table 3).

**Table 3:** Frequency of retinal fluid.

	N of eyes with subretinal fluid		N of eyes with intraretinal fluid	
	before	after	before	after
OG	5	3	9	2
CG	7	4	5	3

**Fig. 3:**
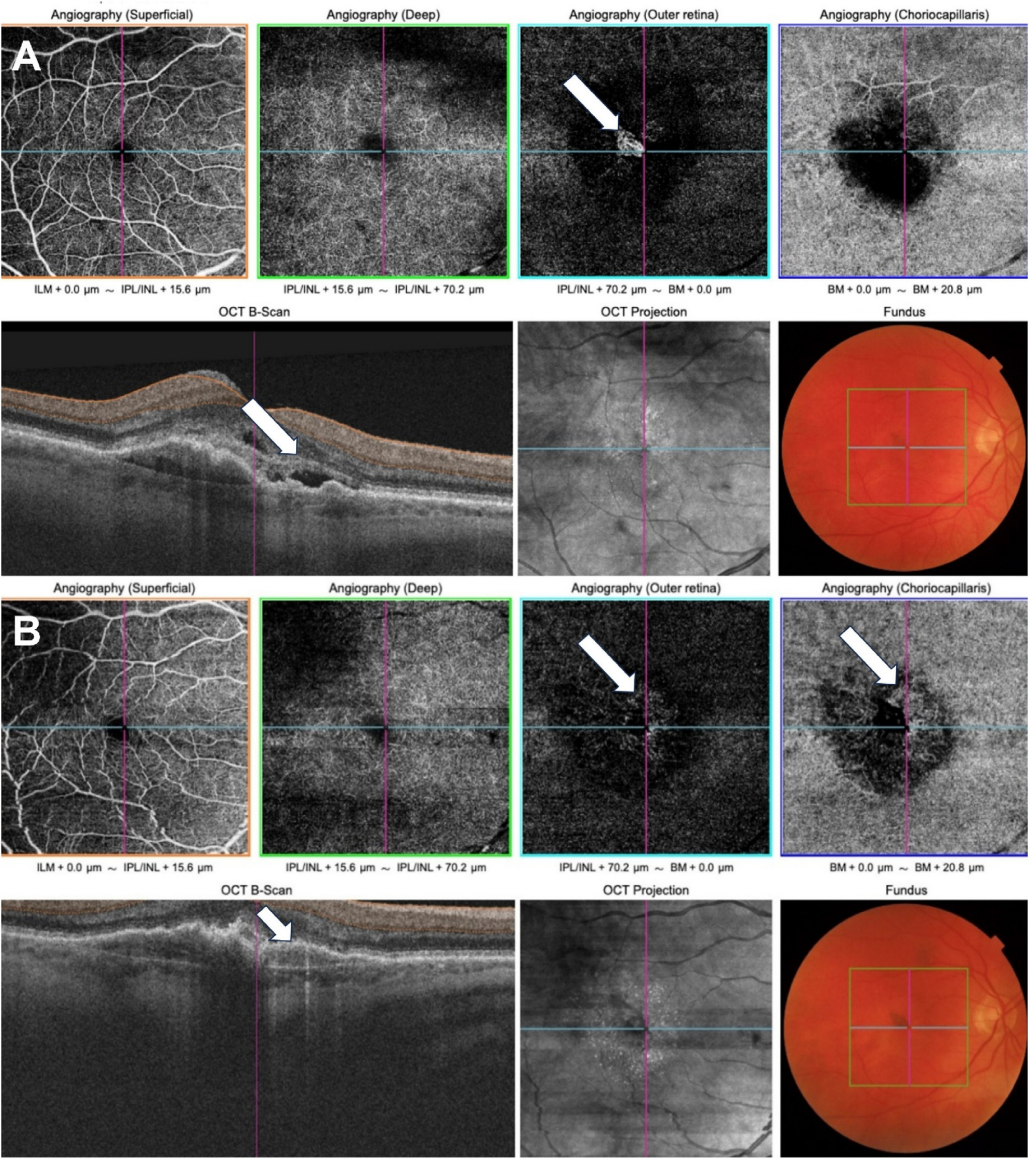
OCT-A scan, OCTB scan and color fundus photo of a study eye at the beginning (A) and the end (B) of the study. (A): The arrow on the OCTA scan points to the NV in the outer retina. The arrow on the OCTB scan points to the presence of intra and subretinal fluid at the beginning of the study. (B): On the OCTA scan after three IVIs and four months of Ocufolin, the arrow indicates where the regression of the NV in the outer retina has taken place. The arrow on the choriocapillaris points to a dense capillary network and anastomosis indicating an increase in blood flow. The arrow on the OCTB scan points to the place where the intra and subretinal fluid has disappeared.

As an illustration, we show an example with a morphologically favorable evolution of an eye of a patient in the OG (Fig. 3).

## 4 Discussion

The treatment of AMD, particularly nAMD, has made great progress, especially with the introduction of anti-VEGF injections.^30^^,^^32^ Nevertheless, this is not yet an ideal treatment or even a definitive cure. Patients with nAMD often have elevated Hcy levels. Our study shows that the additional administration of Ocufolin along with anti-VEGF therapy reduces elevated homocysteine levels in the blood to a statistically significant degree. Is this clinically relevant or are we just correcting an epiphenomenon? Older studies would suggest that we may only have improved a laboratory parameter, but not the course of the disease. Today, however, we know that an elevated Hcy level is not only a useful parameter for a disturbed C1 metabolism, but also has a damaging effect itself.^33^ In many earlier studies focusing on decreasing the Hcy level, folic acid was administered. However, folic acid must first be converted into the biologically active L-5-methylfolate in our body, via several steps. This requires the enzymes DHFR (dihydrofolate reductase) and MTHFR (methylenetetrahydrofolate reductase). In the meantime, we have learned a) that the capacity for folic acid conversion in humans is much smaller than one might assume based on animal experiments^34^ and b) that many people have a DHFR polymorphism, meaning they display even weaker activity.^35^ This not only means that the desired effect is not sufficiently achieved, but also causes an undesirable increase in unmetabolized folic acid (UMFA) in the body.^36^ Although L-methylfolate is the most important component of Ocufolin, it is not the only one. The interaction with all the other components, especially with vitamin B12, is probably decisive.^37^ One pathogenetically important factor in nAMD is oxidative and nitrosative stress. Data from the literature show that the retina with AMD has an increased response to oxidative stress compared to the normal ageing retina. In animal models, studies have shown that vitamins and antioxidant supplements can preserve the retinal pigment epithelium and influence proteins involved in oxidative stress pathways.^38^ L-methylfolate has an antioxidative and an antinitrosative effect.^39^ Furthermore, our study shows that Ocufolin addition along with anti-VEGF therapy reduces elevated RVP levels to a statistically significantly greater extent than anti-VEGF therapy alone. It has already been shown that RVP has a major long-term influence on the progression of glaucoma.^40^ There is much to suggest that this is also the case with AMD, but this was not the subject of this study. The mechanism by which Ocufolin reduces the RVP is not definitively understood. The following may play a role: the reduction in Hcy reduces the expression of the endothelin type A receptor (ETRA) in vascular smooth muscle cells,^41^ which is relevant because ETRA is expressed in the vascular smooth muscle cells of the retinal veins.^42^ Ocufolin reduces oxidative stress in various ways, e.g., by scavenging peroxynitrite through L-methylfolate,^43^ and this is also important in this context because oxidative stress stimulates the expression of endothelin^44^ and endothelin increases the RVP.^8^ This is clinically relevant because a high RVP lowers the PP and, thus, also the blood flow. The resulting hypoxia stimulates the expression of VEGF and proinflammatory molecules. A high RVP also increases the transmural pressure in the retinal capillaries. This leads to increased fluid extravasation. This is particularly unfavorable, since the retina has no lymphatic drainage and any accumulation of fluid leads to a functional impairment. We can assume that Ocufolin also lowers the venous pressure in the choroid (not measured), because the vortex veins are regulated in a similar way to that in the retinal veins.^45^

The clinically indicated anti-VEGF therapy was administered according to a fixed schedule. The additional reduction of homocysteine and RVP by Ocufolin showed a favorable influence on the resorption of retinal fluid in our patients. However, before we can make a general scientific statement, this must be verified with larger case numbers and quantitative analyses.

In our population, patients who had received Ocufolin needed VEGF treatments less frequently afterwards. Although this is an interesting observation, this effect has not yet been scientifically proven.

L**imitations**: All study participants were white Europeans. Thus, these results cannot be generalized to other ethnic groups. The study was not double-blind and so we cannot rule out a certain placebo effect of Ocufolin. The number of patients was small and the duration of the study was short. In addition, all patients already had a long history of anti-VEG therapy. Starting Ocufolin administration earlier in the course of the disease and administrating it for longer may have a prophylactic effect on dAMD and may also delay the transformation of dAMD into nAMD and/or GA.

## 5 Conclusions

Despite the small number of patients and the short duration of the study, Ocufolin was shown to statistically significantly reduce both Hcy and RVP levels. There is much to suggest that lower Hcy and RVP levels could improve the prognosis of nAMD. The observed favorable influence on morphology, and treatment intervals for anti-VEGF therapy also supports this assumption.

## Study approval

The authors confirm that any aspect of the work covered in this manuscript that involved human patients or animals was conducted with the ethical approval of all relevant bodies and the study was performed in accordance with the Declaration of Helsinki，and the protocol was approved by the Ethics Committee of the Eye Clinic Orasis, Department for Medical and Surgical Retina, Reinach, Switzerland.

## Author contributions

The authors confirm contribution to the paper as follows: Conception and design of study: JF, TJ; Data collection: TJ; Analysis and interpretation of results: TJ, JF, AS; Drafting the manuscript: TJ, JF, KK; AS; All authors reviewed the results and approved the final version of the manuscript.

## Funding

This research did not receive any specific grant from funding agencies in the public, commercial, or not-for-profit sectors.

## Declaration of competing interest

The authors declare that they have no known competing financial interests or personal relationships that could have appeared to influence the work reported in this paper.

## Abbreviations

AREDS: Age-Related Eye Diseases Study
BCVA: Best Corrected Visual Acuity
CG: Control Group
dAMD: dry Age-related Macular degeneration
ETRA: Endothelin Receptor A
GA: Geographic Atrophy
Hcy: Homocysteine
ICP: Intracranial Pressure
IOP: Intraocular Pressure
IVI: Intravitreal Injections
nAMD: neovascular Age-related Macular Degeneration
NV: Neovascularization
OCT: Optical Coherence Tomography
OCTA: Optical Coherence Tomography Angiography
OG: Ocufolin Group
PP: Perfusion Pressure
RVP: Retinal Venous Pessure
VEGF: Vascular Endothelial Growth Factor
